# Osteomyelitis of the mandible after insect bite in a pediatric patient

**DOI:** 10.1590/0037-8682-0165-2023

**Published:** 2023-06-02

**Authors:** Uluhan Eryuruk, Ismet Mirac Cakir

**Affiliations:** 1 Giresun University, Faculty of Medicine, Department of Radiology, Giresun, Turkey.; 2 Samsun Education and Research Hospital, Department of Radiology, Samsun, Turkey.

**FIGURE 1:**
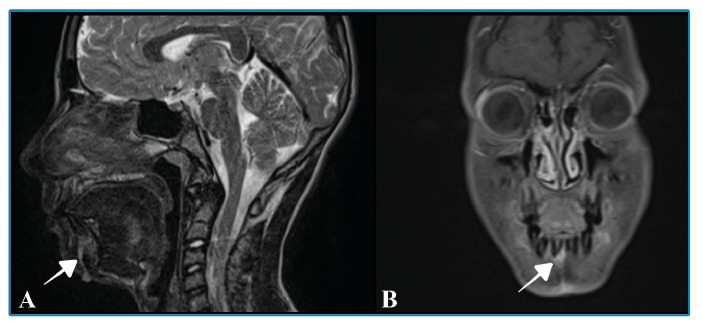
A: Sagittal T2-weighted magnetic resonance (MR) images showing a sinus tract (arrow) crossing the mandible and opening into the skin, a manifestation of osteomyelitis. B: Coronal fat-supressed contrast-enhanced T1-weighted MR images showing enhancement in the mandibular corpus at the alveolar level.

A 13-year-old boy was admitted to our hospital with a complaint of jaw swelling. He reported being bitten by an insect, the Dracula beetle, during hazelnut harvesting 2 months prior to admission. He had no history of atopy or previous insect bite sensitization. Despite topical treatment, the swelling increased progressively. Magnetic resonance imaging revealed a sinus tract crossing the mandible corpus and opening into the skin (Figure 1A). After intravenous administration of the contrast agent, enhancement of the corpus mandibula was observed at the alveolar level (Figure 1B). Based on these imaging findings, mandibular osteomyelitis was suspected. Blood cultures were negative; however, the wound culture revealed methicillin-resistant *Staphylococcus aureus*.

*Anoplophora chinensis,* or the Dracula beetle, is an invasive insect whose larvae feed on the lower trunks and exposed roots of many tree species in forested habitats. No toxicity or irrigation effects of *Anoplophora spp.* have been reported in humans^1^. Arthropod bites are common in pediatric patients, and most cases are benign and self-limiting. Rare cases of osteomyelitis caused by arthropod bites have been reported, with only one case of mandibular osteomyelitis reported in an adult patient^2^ ^,^ ^3^. In such cases, the pathogenesis of osteomyelitis is attributed to bite-related traumatic injuries, which facilitate bacterial infection^3^. Similarly, in our patient, we believe that microtrauma and local inflammation of the skin after the beetle bite predisposed the patient to bacterial infection, leading toosteomyelitis. Clinicians should be aware of the serious complications, such as mandibular osteomyelitis, following insect bites in pediatric patients.
